# Observations on Several Pharmaceutical Preparations

**Published:** 1804-02-01

**Authors:** 

**Affiliations:** Druggist, at Paris


### *Observations on several Pharmaceutical Preparations, by* Cit. Steinacher, *Druggist, at Paris.*

### *Unguentum Nutritum.*

Citizen Dubree, an eminent druggist at Rouen, has lately presented a formula for unguentum nutritum, to the Pharmaceutical Society. As apothecaries, zealous for the perfection of their art, have proposed improvements in the preparation of this ointment at different periods, I have thought that an object, to which the attention of practitioners has been called from time to time, notwithstanding it is apparently obsolete, deserved a fresh examination.

When oil, vinegar, and litharge are to be mixed together into a homogeneous mass, a little litharge must be dissolved in acetous acid, and a sufficient quantity of carbonic bonic acid must be introduced. 1st. to convert the greater part of the litharge into white carbonate, which remains diffused through the oil; 2dly, to thicken the oleous mixture, an effect analogous to the thickening of soups by the carbonic acid, with which we were made acquainted by Pelletier. If a sufficient quantity of vinegar to form a saturated saline compound be employed, the mixture will never combine perfectly. This theory, founded on experiment, brings us back to the prescribed formula, as the best that can be adopted, that which produces an ointment, the most bulky, the lightest, and the most cooling to the part affected. It succeeds very well, when the operator is endued with patience, and works in a cold place. It may be abridged however, if, according to the excellent advice of Baumé, we employ coagulated oil of olives, by which the surfaces of contact are increased, and the introduction of the air is facilitated. One important fact with respect to keeping the preparation is, that at the temperature of 15° or 16°, at which most kinds of fermentation take place, a portion of the carbonic acid is extricated, and leaves exposed an oxyd at $1\frac{9}{100}$, which becomes again yellow. It requires a temperature of 10° to preserve its white colour unaltered.

Citizen Dubree, and Citizen Granet before him, proposed to expedite the preparation by adding hog's lard; but I find, that this addition diminishes its bulk and levity. In Germany, different compositions are made under the name of *nutritum*, as with vinegar of litharge and half its weight of oil of roses, which produce an ointment as white as wax, and of the consistence of a liniment; with vinegar of litharge two parts, and olive oil three parts, which yield a whitish ointment of a moderate consistence; with two parts of olive oil, one part of wax, and two parts of vinegar of litharge, which furnish an ointment of a firm consistence, and a waxy whiteness. But all these compositions are simple mixtures, feebly united, by no means resembling the nutritum of the French shops, and not requiring for their formation a mutual reaction between the different particles of the ingredients.

### *Crystallization of Phosphoric Acid.*

It is known, that the affinity of phosphoric acid for water overpowers its force of crystallization; in fact, this salifiant substance appears commonly in the form of a thick oil. I have lately observed, however, that time, the grand producer of regular crystallization, effects a symmetrical combination between its particles.

I had prepared half a kilogramme of phosphoric acid, according to the method of Lavoisier, with phosphorus and nitric acid, both of them extremely pure. This acid, freed from nitrous gas, reduced to the consistence of a thick syrup, and introduced into a phial with a glass stopper, had been used at different times in the course of a year, without exhibiting any peculiar appearance. The year following I let it remain perfectly at rest in the phial, which was half full, and closely stopped. After this period I found the surface of the fluid covered with a saline crust, from which shot downward prismatic crystals in shining laminæ, an inch long, and a line broad, diverging from a centre. I will not describe their geometrical structure, for they are extremely thin, and embedded in a fluid too viscous for me to take them out without breaking. Besides, they are still increasing; laminæ rise from the bottom of the vessel, which touch the surface of the glass, and seem preparing to intermix with the ramifications that shoot down from the upper stratum. The sides of the vessel are the seat of this beautiful crystallization. The centre remains in part concrete, or fluid, whence it follows, that if a very regular dissipation of the particles of the liquid acid of phosphorus be occasioned by repose, the sides of the vessel contribute to it in great measure by affording fixed points, to which the positions of affinity most favourable to crystallization direct themselves.

### *Purity of Phosphorus.*

Proust has informed the public, that, in the distillation of phosphorus, a combination of this substance with the charcoal constantly took place. This important discovery extends much farther than its celebrated author has shewn.

Take the most brilliant and most transparent phosphorus, which has not only been strained through chamois leather, according to Woulfe's method, but has also been dissolved several times in nitro-muriatic acid, as done by Count Mussin-Puschkin, or which has been treated with oxygenated muriatic acid, after the mode of Juch of Wurkburg, let it be heated gently in a long slender tube; red parts will separate from it Put a few grains of this phosphorus, which is conceived to be so pure, on a silver spoon, and set fire to it; a red trace will remain. If the spoon be heated in in the dark, the red trace will be seen still to burn, and a coal will remain impregnated with phosphoric acid.

Mr. Juch has asserted, that his phosphorus is extremely pure, because it no longer becomes black when heated with caustic alkali; but it is in fact because the phosphure of carbon is unalterable by caustic potash. According to the indisputable authority of Proust, this re-agent is incapable of proving the purity of phosphorus. I confess, that heated oxygenated muriatic acid destroys part of the carbon of phosphorus, because the combustible power of its oxygen increases in the ratio of its elasticity; but it produces this effect only by burning a proportionate quantity of phosphorus. On the contrary, when it is cold, and its oxygen is reduced to its natural degree of elasticity, it is far from destroying the carbon; it separates it in the state of black oxyd, and converts the phosphorus into white oxyd, while at the same time, itself returns to the state of simple muriatic acid. I have observed this fact on a stick of transparent phosphorus, which I kept two years in a bottle filled with pure oxygenated muriatic acid, saturated at the temperature of 10°. It is impossible, therefore, to free the phosphorus entirely of charcoal. They oxyde, or are acidified nearly in proportional quantities; and though the proportion of charcoal may be diminished, the phosphorus always retains some by its power as a whole. In fine, I am obliged to contradict the assertion of an illustrious master, Citizen Fourcroy, "that we are unacquainted with any direct combination between carbon and phosphorus, though it probably exists," and to consider that product on which chemists have hitherto bestowed the name of pure phosphorus, as a kind of gangue, from which the radical phosphorus is disengaged to enter into a number of combinations, without our being capable of obtaining it in its primitive form.

*White Oxyd of Phosphorus.*

When phosphorus is heated in a very long and very slender glass tube, in a sand-heat of 100° of the decimal thermometer, it is covered with a mild light, and exhales a white vapour, which condenses in the upper part of the tube, while, at the same time, part of the phosphorus, with excess of carbon, separates with its red colour. This white vapour, which has acquired for its formation a slight combustion, is a white oxyd of phosphorus at a minimum. The following are some of its properties. It is flocculent, possessed of cohesion, and occupies four times the space of the phosphorus employed in the experiment. When it is dry, it does not redden litmus paper. It contains caloric and inflames on the contact of combustible substances. It powerfully attracts the moisture of the air, and is rapidly converted into phosphorus acid. It differs greatly from the white oxyd of phosphorus made by the long action of water, or cold oxygenated muriatic acid. This appears friable and pulverulent. It has lost almost all its latent heat. It is very little inflamable, and does not attract the moisture of the air. It is acidifiable only by the intimate action of an oxygen that contains caloric highly condensed, as that of the nitric acid. In a word, it is phosphorus at a maximum of oxydation.

*Regular Crystallization of Essential Oil of Roses.*

Citizen Steinacher has lately observed this with attention. He mixed eight kilogrammes of the magna of damask roses (*roses pâles*) with some parts of water, according to the process of Cit. Demachy; and after a day's maceration he drew off by distillation sixteen kilogrammes of water. This was immediately poured into a large glass jar, which was covered with a cloth, and left at rest. In twenty-four hours he found the surface of the water covered with an irridescent pellicle, interspersed with hexaedrons, very much resembling the crystals of snow, which the illustrious Cit. Mongé has described. He informs us, that a slight shake is sufficient to tear the crystalline gauze, and reduce it to that irregular form of whitish scales or laminæ, which the oil of roses commonly assumes.

